# Does Waiver of Informed Consent in Low-Risk Randomised Controlled Trials Improve Research Inclusion? Insights from a Review of the UK Health Research Authority Confidentiality Advisory Group Register

**DOI:** 10.1177/15562646261471811

**Published:** 2026-08-03

**Authors:** Alastair Watson, Kane Alexander, Estelle Payerne, Stephen Morris, Jonathan P Fuld, Polly-Anna Ashford

**Affiliations:** 1 School of Clinical Medicine, 2152 University of Cambridge, Cambridge, UK; 2 Cambridge University Hospitals NHS Foundation Trust, Cambridge, UK; 3 Chelsea and Westminster Hospital NHS Foundation Trust, London, UK; 4 Norwich Clinical Trials Unit, 6106 University of East Anglia, Norwich, UK; 5 Primary Care Unit, Department of Public Health and Primary Care, 12204 University of Cambridge, Cambridge, UK; 6 Department of Respiratory Medicine, Cambridge University Hospitals NHS Foundation Trust, Cambridge, UK

**Keywords:** trial design, research inclusion, waiver of informed consent, informed consent, low-risk pragmatic trials

## Abstract

Low-risk pragmatic trials improve clinical services. Informed consent is gold standard to ensure ethical research. However, its implementation can impact participation of underserved groups who face disproportionate barriers around communication, health and research literacy, and limited availability for burdensome processes. Carefully considered application of waived consent may overcome barriers to inclusive participation. However, an understanding of when waived consent has been used, whether it supported inclusion research, and lessons that can be learnt, is lacking. We reviewed the UK Health Research Authority Confidentiality Advisory Group (HRA CAG) database for non-emergency individually randomised trials that were granted a waiver of consent. Nine trials were identified; these aimed to boost cancer screening uptake in deprived or underserved populations. Waived consent enabled inclusive research approaches for evaluating interventions such as culturally tailored screening kits and barrier-reducing tools. Further research is needed on patient perspectives and ethical safety in these consent models.

## Background

Low-risk pragmatic research, including randomised trials, is becoming increasingly important for developing clinical services and improving patient outcomes. Informed consent is a core principle of Good Clinical Practice and ensures the autonomy and safety of patients (General Medical Council, 2024). However, the process of informed consent, in which patients are typically asked to read and understand an information sheet, attend a study location, and initial and sign a consent form with a researcher, can be burdensome and lead to the disproportionate exclusion of certain groups with additional barriers to healthcare or research (Brandon & Bagnall, 2024; Busisiwe et al., 2023; McDonald et al., 2024; Mohan & Freedman, 2023; Peters et al., 2023). Such selection and self-selection (volunteer) bias can occur due to barriers such as limited health and research literacy, low income, inflexible employment, caring responsibilities, and even investigator bias (Lewis et al., 2003; Mitchell & Kline, 2008). Ethnicity is also a key dimension of health inequalities and research participation, with ethnic minority groups experiencing poorer health and health-related quality of life, and higher incidence of long-term conditions, alongside higher rates of unemployment and lower income than White British households (Byrne et al., 2020; Dharni et al., 2017; Moser et al., 2009; Office for National Statistics [ONS], 2022; Pardhan et al., 2025). Consequently, we risk having an incomplete understanding of the safety and efficacy of new interventions when they are rolled out to the general population.

Furthermore, the evaluation of interventions that are fundamentally designed to change behaviour and increase patient understanding and engagement have the potential to be significantly influenced by pre-intervention engagement in research activity such as informed consent, leading to unreliable findings. Conversely, a waiver of consent describes situations where investigators are not required to request permission from research participants prior to enrolment; this may mean that participants are informed of the trial and given the opportunity to opt out, but are not required to ‘opt in’; or a full waiver in which participants may not know they are included in a trial.

Ethical solutions to conducting these studies in a way that provides sound evidence from a sample that is representative of real-world populations is essential for improving patient outcomes and tackling disparities in healthcare. Increased awareness of the issue of underrepresentation is leading researchers to explore a multitude of approaches, from community engagement, translated and culturally tailored recruitment materials, reducing the number of in-person study visits, and use of digital and community-informed recruitment strategies (Heffernan et al., 2023; Wieland et al., 2021). The lower barriers associated with waived consent could mitigate these risks and be a more inclusive participation model when applied under carefully considered circumstances.

A recent article by Siriwardana et al. examined the current guidelines available to researchers regarding the circumstances under which a waiver of consent could be ethically acceptable (Siriwardana et al., 2025). The authors demonstrated that the guidelines agreed on three overarching criteria: that the research would not be otherwise feasible, that it has important social value and that it is of minimal risk. This serves as a useful framework to add clarity to the field and support future inclusive research. However, to implement waiver of consent effectively and inform trial design, it is also essential to reflect on lessons from previous studies that have used this approach. An understanding within the literature of the occurrence of waived or deferred consent in UK trials, the reason for that choice of consent model, as well as the context and lessons that can be learnt is lacking. Flory et al. identified that cluster randomized designs and emergency research routinely use a waiver of consent because of the impracticability of individual informed consent in those contexts (Flory et al., 2016). These issues within cluster randomized trials have been discussed and a framework has been offered by Nix et al. who highlight issues of participants not being aware they have been randomised or being able to opt out due to the nature of cluster-level interventions (Nix et al., 2021). However, drawing out examples of waived consent in the more ethically contested arena of individually randomised trials in non-emergency settings is essential to inform the design of inclusive low-risk trials in the future.

Clinical audits and quality improvement projects are fundamental to advancing clinical care and can advance knowledge through evidence generating medicine approaches (Balu et al., 2021; Embi & Payne, 2013; Ozsancak Ugurlu et al., 2026). Nevertheless, additional clinical research is critical in allowing specific aims to be met by testing hypotheses using standardised research methods to produce generalisable results, as highlighted in the Health Research Authority and decision tool (Health Research Authority [HRA], 2024b). We recognise the important role of real-world data studies using existing health data and biological material, governed by international ethical standards such as the Declaration of Taipei (World Medical Association [WMA], 2016). Nevertheless, RCTs add particular value due to the level of evidence they provide for causality which can be used to improve clinicals services. Furthermore, waiver of consent approval is most ethically contested in individually randomised designs within ethics committees where active intervention allocation occurs without participant authorisation. Understanding where waiver of consent is used in these studies and what can be learnt is therefore key and could directly inform use of such approaches in other study types, including cohort studies.

We sought to capitalise on the availability of the UK Health Research Authority (HRA) Confidentiality Advisory Group (CAG) register. The UK HRA CAG register contains UK studies requiring ‘section 251’ support for sharing of patient identifiable data outside the care team without individual informed consent (Health Research Authority, 2026; National Health Service Act. (2006)). Approval by the HRA CAG supports the ‘public interest’ legal basis for processing personal data under the GDPR and complements approval by an NHS Research Ethics Committee. The CAG approval register allows the identification of waived/deferred consent trials, as consent-based trials obtain permission for personal data processing within informed consent and therefore generally do not require CAG approval. We comprehensively searched the UK HRA CAG register to investigate where waiver of consent had been used in UK non-emergency trials, to explore the justification for this approach, and to ascertain if this was related to improving research inclusion.

## Methods

We searched the following CAG application register files using the terms “random” or “RCT”:

“April 2013 onward approved research applications” (covers studies approved from April 2013 onwards)

“January 2009 – March 2013 approved applications (advice provided by the NIGB ECC)”

“2001–2008 approved applications (advice provided by PIAG)”

These filenames refer to different time periods and advisory bodies, as archived by the Confidentiality Advisory Group (CAG). The database “April 2013 onward approved non-research applications” was not reviewed as non-research applications were deemed not likely to contain RCTs (Health Research Authority, 2026). The CAG databased sections were downloaded on 15^th^ February 2025.

We identified and reviewed applications for randomised controlled trials which randomised patients at the individual level and used waiver of consent or deferred consent in non-emergency settings. We defined these as: waiver of consent - where the study received ethical approval which allows the study to proceed without obtaining informed consent; deferred consent - where the study received ethical approval which allows the study to enrol participants and data may be collected before consent is obtained, with consent sought later, often after randomisation (Health Research Authority [HRA], 2024a). The CAG applications were reviewed in full, 15^th^ February to 28^th^ February 2025, particularly for key terms such as “opt out”, “consent”, “waiver of consent” or “deferred consent” as well as for any information that allowed interpretation of the consent process, as well as for any insights about target populations or aims which were relevant to improving inclusivity in research.

Cluster randomised trials, non-randomised studies, or studies which took consent before randomisation for all arms of the study were not included. CAG applications were then reviewed to understand the context of waived or deferred consent and their use for delivering inclusive research and the type of interventions typically associated with this consent strategy. CAG reference numbers, IRAS and REC reference numbers, CAG application names and CAG application titles were also used to search PubMed and Google Scholar to identify publications related to the CAG register trial application, performed on 7^th^ April 2025.

## Results and Insights

**Figure 1.**
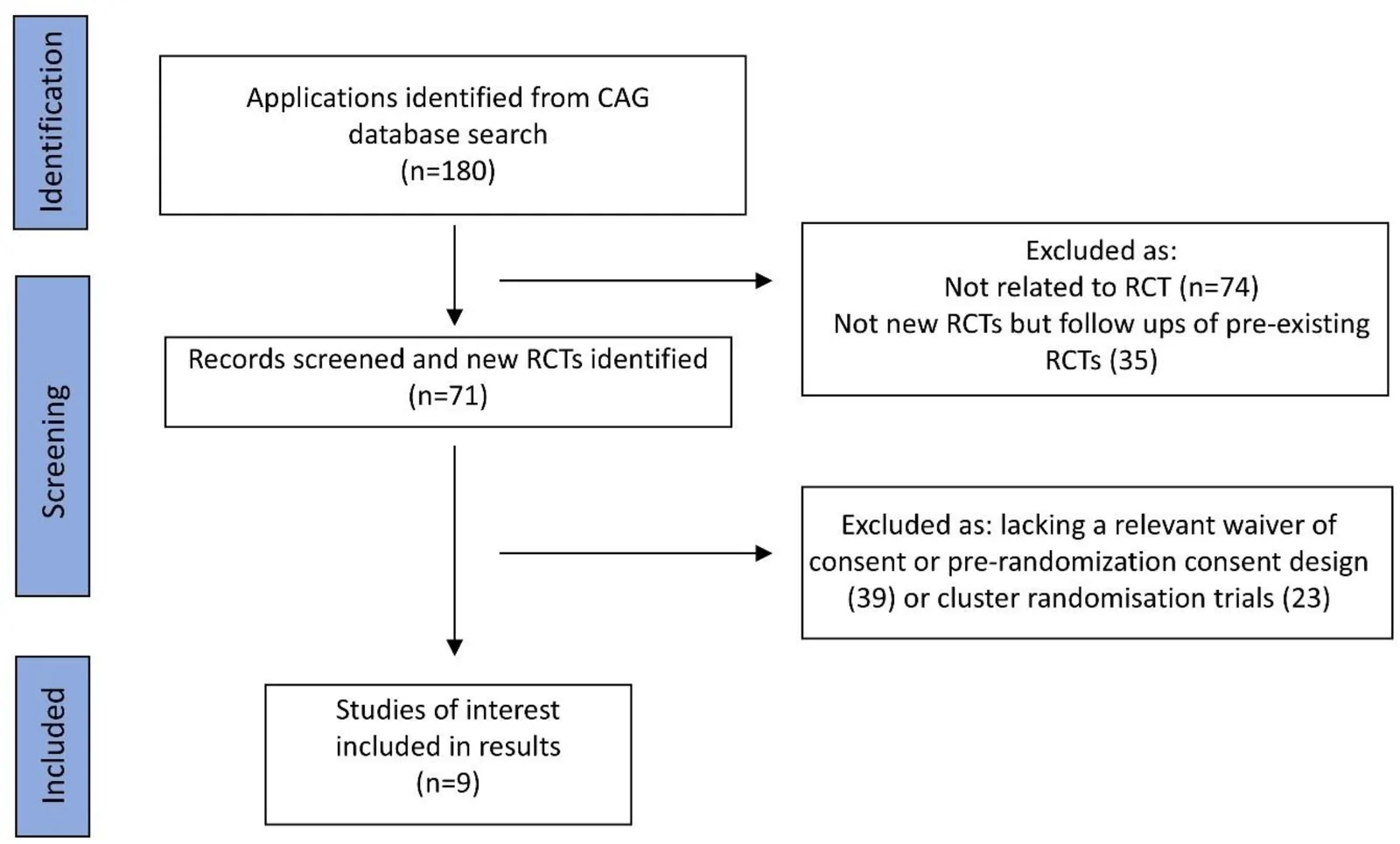
Flowchart of search results and application selection.

180 applications were identified and screened in full and nine relevant applications were identified (Figure 1 and Table 1). The studies all comprised low-risk RCTs aiming to affect behaviour change to increase cancer screening uptake. Eight of the studies used waiver of consent and one study used deferred consent (post-randomisation). Four of these studies had reported primary results (Crosbie et al., 2022; Hirst et al., 2017; Kaushal et al., 2020; Wilding et al., 2023). Five studies had published protocols (Crosbie et al., 2020; Duffy et al., 2024; Hirst et al., 2016; McGregor et al., 2018; von Wagner et al., 2018), two studies had published protocols only with no published results (Duffy et al., 2024; McGregor et al., 2018), and two had only published ISRCTN registrations (Davidson & Carter, 2025; Nichol & Graham, 2024). One study had no associated publications.

**Table 1.** Included studies from the CAG Register.

	Year CAG Register No.	Title	Publication	Consent Model	Population/Target Demographic	Intervention	Target Trial Outcome
1	2017 17/CAG/0162	Use of a GP-endorsed non-participant reminder letter to promote uptake of bowel scope screening	Article: Kaushal et al., 2020 (Kaushal et al., 2020) Protocol: (von Wagner et al., 2018)	Waiver of informed consent.	Non-responders to initial BSS invitation. Hard -to-reach London population with low screening engagement.	GP endorsement added to reminder letter	Uptake of Bowel Scope Screening (BSS)
2	2018 18/CAG/0038	Community-based LDCT screening for lung cancer vs usual care	Article: Crosbie et al., 2022 (Crosbie et al., 2022) Protocol:(Crosbie et al., 2020)	Pre-consent randomisation (Zelen design). Opt out rates: 0.14% of 89 917.	55–80 year old smokers or ex-smokers at increased risk. Deprived area of Leeds	Invitation to community-based Lung Health Check (incl. LDCT)	Feasibility and utility of community-based lung screening
3	2021 15/CAG/0156	Text-message reminders for CRC screening (TRICCS)	Article: Hirst et al., 2017 (Hirst et al., 2017) Protocol: (Hirst et al., 2016)	Waiver of informed consent. Used existing patient messaging service, no new consent gained.	60–74 year olds, non-responders to CRC screening in London	GP text-message reminder at 7 weeks post-kit	Return of FIT kit within 18 weeks
4	2021 21/CAG/0021	Behavioural interventions to increase cervical screening uptake in Yorkshire	Article: Wilding et al., 2023 (Wilding et al., 2023)	Waiver of informed consent	25–64 year old individuals receiving cervical screening invitation (NE & Yorkshire)	Implementation intention, motivational, both, or usual care	Uptake of cervical cancer screening within 16 weeks
5	2020 18/CAG/0049	Primary care interventions to increase bowel scope screening in Yorkshire	Protocol: McGregor et al., 2018 (McGregor et al., 2018)	Waiver of informed consent	Individuals due to receive NHS BSS invitation from Hull and Yorkshire; underserved areas with historically low BSS uptake	Primer letter + self-referral or patient navigation call to address personal barriers	Uptake of Bowel Scope Screening (BSS)
6	2023 22/CAG/0140	Text-message reminders and patient navigation to improve FIT uptake	Protocol: Duffy et al., 2024 (Duffy et al., 2024)	Waiver of informed consent	Adult non-responders to FIT screening in London	Text-message reminders and/or telephone patient navigation	Uptake of FIT screening within 11 weeks
7	2024 24/CAG/0051	SMS reminders with link to decision tool for cervical screening in people with SMI	Registration only: ISRCTN12558681	Waiver of informed consent	People with severe mental illness (SMI) overdue cervical screening, London	SMS with or without link to decision support tool	Feasibility and uptake of cervical screening
8	2024 24/CAG/0070	ACES At Home: Urine test to improve cervical screening uptake	Registration only: ISRCTN58902324	Waiver of informed consent	Women and people with a cervix overdue for cervical screening, Greater Manchester	Mailed urine/vaginal self-sampling kits	Return rate of self-sampling kits and screening uptake
9	2024 24/CAG/0101	Improving Bowel Cancer Screening Uptake in South Asians in Bradford	Not yet published	Waiver of consent	South Asian adults, a group with historically low screening rates, who are due bowel screening in Bradford	Culturally tailored kit: enhanced instructions + biodegradable catcher	Uptake of bowel cancer screening

Unless otherwise stated, interventions listed are the intervention plus standard of care compared with standard of care only.

The studies were all aiming to reach participants from either deprived areas or underserved communities, invitation non-responders or communities with previously low response rates (Table 1). All studies evaluated a non-clinical intervention underpinned by behaviour-change techniques (Michie et al., 2018), designed to increase the uptake of standard clinical care, namely lung, bowel or cervical cancer screening as their target outcomes. Intervention strategies to improve screening uptake included a culturally tailored enhanced screening kit for South Asians in Bradford. Other studies used tools to address either personal barriers or barriers associated with severe mental health conditions, including motivational letters, reminders and patient navigation calls.

In a study of GP reminders for bowel scope screening non-participants, Kaushal et al. report the recruitment of a sample of 1200 participants that was well balanced across Index of Multiple Deprivation (IMD) quintiles (Kaushal et al., 2020). IMD quintiles divide England into five equal population bands (20% each) based on deprivation rank, with quintile 1 representing the most deprived 20% nationally (Ministry of Housing Communities & Local Government, 2019). Studies apply and use quintiles using region-specific IMD data but are nationally comparable. A large sample size is a common theme in the identified studies, with Wilding et al. reporting access to a sample of 14,466 participants with eligible data for analysis in a cervical cancer screening trial, 33% of which were from the most deprived IMD quintile (Wilding et al., 2023). Similarly, the TRICCS study included 8,269 participants, 21% from the most deprived IMD quintile (Hirst et al., 2017). Unfortunately, these three studies were unable to report on the ethnicity of participants, having limited access to demographic data.

The largest published trial, the Yorkshire Lung Screening Study, randomised 89,917 participants (Wilding et al., 2023). 50% of the intervention group were reported as White and 41% as Mixed ethnicity. Crosbie et al. describe this study as a Zelen design, a design where patients are randomised to intervention and control before seeking informed consent, with the intervention arm only being approached for consent to their allocated group, whilst the control receives usual care without providing research consent (Zelen, 1979). In the Crosbie study only the participants in the intervention arm who went on to attend the lung health check itself were given a full explanation of the research study alongside information about the harms and benefits of the lung screening procedure. This is also the only published study to report on participant opt-out rate, with just 0.14% of the 89,917 participants excluded from the analysis due to having registered dissent either during the study or via the NHS National Data Opt-Out. None of the studies reported patient perceptions of the consent waiver or opt-out participation approach of the trial.

## Discussion

We have identified an emerging use of consent waivers to improve trial inclusivity or maximise the participation of underrepresented groups. In the CAG-approved studies we found the interventions being tested were non-clinical, supporting uptake of evidence-based standard care in the NHS. Neither the study intervention nor data sharing posed significant risks to participants, and approval by the HRA CAG indicates independent agreement that a consent waiver was appropriate and proportionate to the low-risk nature of the research. The studies identified are therefore consistent with the criteria laid out in the legal frameworks highlighted by Siriwardana et al. regarding minimal risk research of high social value that would otherwise be unfeasible or impractical.

The extremely low opt-out rate reported in the Yorkshire Lung Screening Study suggests that most patients are either supportive of, or at least not actively opposed to, an opt-out consent model, though the evidence is limited and it is important to bear in mind patient understanding of or accessibility to the opt-out process which could impact this. In the published trial results, we observe that the consent waiver provided researchers with a patient sample that was sufficient in size and demographically appropriate to answer their hypothesis. This would have been impossible to achieve via informed consent due to the infeasibility of obtaining consent from such a large population for which the rate of consent may only be 5–15% of those approached, and the high likelihood of selection bias undermining the validity of the findings.

Fundamentally, the waived consent, or deferred consent could be a powerful tool to improve the representativeness of trial populations, but this consent approach is a highly active topic of discussion among trialists globally and important questions remain to be answered (Baker & Merz, 2018). These include: how widespread is the use of waivers of consent in non-emergency trials? Are safety and ethical concerns being fully and consistently addressed by researchers? What can we learn from previous studies about the impact of waiver of consent participation on trial populations? We have been unable to identify any reports on patient views of waiver of consent in non-emergency interventional research. It is important to establish whether there are communities who find a waiver of consent with opt-out model less acceptable for example because of cultural considerations, or current or historic issues around medical and research mistrust.

A key ethical tension underpinning the opt-out trials we identified is the apparent paradox between promoting inclusion and modifying or waiving prospective informed consent. This raises legitimate concerns regarding respect for autonomy and risks framing consent as a “barrier” rather than a fundamental participant right. Rather than positioning waiver of consent as a substitute for participant involvement, we argue it should be understood as one component which requires careful consideration as part of a broader strategy to enable fair access to research participation, particularly where traditional consent processes systematically exclude underserved populations.

In this context, the principle of meaningful engagement is critical. Inclusive research must remain grounded in the principles of patient and public involvement, in which research is undertaken “with or by” the public rather than “to, about or for” them, reflecting the broader ethos of meaningful engagement in research design and delivery (Boote et al., 2015; Staniszewska et al., 2017). For studies using opt-out or waived consent models, this must include early community engagement, culturally appropriate communication strategies, transparency about data use, and accessible mechanisms for dissent.

Our pragmatic search approach was limited to the HRA CAG register and will therefore only contain UK trials where patients’ identifiable data has been shared outside the care team. This approach allowed us to rapidly identify trials of interest, particularly trials that could raise additional confidentiality concerns for patients, but does not include trials without informed consent if no sharing of identifiable data was required, as these studies would require only standard ethical approval. As only four of the nine identified studies have published results, we can discern relatively few insights about safety and ethical considerations, acceptability to patients and the impact of waived consent on study findings. Further research is now essential to generate understandings about whether such models could improve the inclusion of individuals who are often under-represented in research due to socioeconomic barriers, while appropriately addressing ethical and safety considerations.

## Conclusion

A waiver of consent has traditionally been employed in emergency settings where consent could not be sought prior to enrolment, and in large-scale cluster randomised trials where it is important to understand the impact of an intervention on all patients who would be exposed to it, such as service improvement strategies. Through our search, we illustrate that waiver of consent models are also being used to undertake low-risk randomised controlled trials with a focus on inclusivity and underserved participant groups. Further work to understand the tangible benefits, drawbacks and participant acceptability of using a waiver of consent or other alternative model to full informed consent is required.
